# Hybrid Polyelectrolyte Capsules Loaded with Gadolinium-Doped Cerium Oxide Nanoparticles as a Biocompatible MRI Agent for Theranostic Applications

**DOI:** 10.3390/polym15183840

**Published:** 2023-09-21

**Authors:** Danil D. Kolmanovich, Nikita N. Chukavin, Irina V. Savintseva, Elena A. Mysina, Nelli R. Popova, Alexander E. Baranchikov, Madina M. Sozarukova, Vladimir K. Ivanov, Anton L. Popov

**Affiliations:** 1 Institute of Theoretical and Experimental Biophysics, Russian Academy of Sciences, Pushchino 142290, Russia; 2 Kurnakov Institute of General and Inorganic Chemistry, Russian Academy of Sciences, Moscow 119991, Russia

**Keywords:** cerium oxide nanoparticles, gadolinium, polyelectrolyte microcapsules, MRI agent

## Abstract

Layer-by-layer (LbL) self-assembled polyelectrolyte capsules have demonstrated their unique advantages and capability in drug delivery applications. These ordered micro/nanostructures are also promising candidates as imaging contrast agents for diagnostic and theranostic applications. Magnetic resonance imaging (MRI), one of the most powerful clinical imaging modalities, is moving forward to the molecular imaging field and requires advanced imaging probes. This paper reports on a new design of MRI-visible LbL capsules, loaded with redox-active gadolinium-doped cerium oxide nanoparticles (CeGdO_2−x_ NPs). CeGdO_2−x_ NPs possess an ultrasmall size, high colloidal stability, and pronounced antioxidant properties. A comprehensive analysis of LbL capsules by TEM, SEM, LCSM, and EDX techniques was carried out. The research demonstrated a high level of biocompatibility and cellular uptake efficiency of CeGdO_2−x_-loaded capsules by cancer (human osteosarcoma and adenocarcinoma) cells and normal (human mesenchymal stem) cells. The LbL-based delivery platform can also be used for other imaging modalities and theranostic applications.

## 1. Introduction

Medical imaging has long served as an important tool for diagnosis and therapeutic efficacy monitoring. MRI is safe and has a very high spatial resolution of around 25–100 μm in different magnetic fields. Today, gadolinium-containing compounds are widely used as MRI contrast agents [1]. The Gd^3+^ ion has seven unpaired electrons (_8_S^7/2^) and an exceptionally high magnetic moment (7.94 BM), which enables the use of gadolinium-containing compounds as contrast agents in magnetic resonance imaging (MRI). Gadolinium in ionic form is very toxic and can potentially cause nephrogenic systemic fibrosis (NSF) [2,3]. In this regard, various chelating compounds are used, for example, dipyridoxal phosphate, 1,4,7,10-tetraazacyclododecane-1,4,7,10-tetraacetic acid, diethylenetriaminepentaacetic acid, etc., which limit its direct contact with biological fluids. This principle is implemented in all known clinical preparations based on gadolinium (Omniscan^®^, OptiMark^®^, MultiHance^®^, Primovist^®^, and Vasovist^®^). At the same time, poorly soluble gadolinium oxide is a more stable compound than its ionic form and does not have toxic effects either in vitro or in vivo [4,5]. Gadolinium oxide in nanocrystalline form is considered one of the most promising contrast agents for MRI, due to its higher value of longitudinal relaxation constants in comparison with Gd^3+^ chelate complexes [6].

The cerium oxide nanoparticle (nanoceria, CeO_2_) is the most promising inorganic nanozyme, and it is used in various areas of biomedicine. It has strong antioxidant [7], radioprotective [8], and anti-inflammatory properties [9]. At the same time, the doping of cerium oxide with Gd^3+^ ions increases its oxygen nonstoichiometry and, consequently, enhances the antioxidant activity of the nanoparticles [10], and also increases T1 relaxivity. Thus, gadolinium-doped cerium oxide nanoparticles are a good ground for the design of new theranostic agents, which possess both high redox activity and MRI contrasting ability.

It has been shown recently that nanoceria is capable of exhibiting selective cytotoxicity against cancer cells [11,12,13], but the ultrasmall particle size of bioactive nanoceria and its high reactivity limits the effective targeted delivery to tissues and organs. In this regard, there is a need to create advanced nanoceria delivery systems providing the concentration and delivery time control. One possible solution to this problem is the use of polyelectrolyte microcapsules. Polyelectrolyte capsules are constructed through layer-by-layer (LbL) self-assembly techniques and have been shown to be a promising platform for various biomedical applications [14,15,16,17,18,19,20]. The advantages of LbL capsules include the ease of their size, chemical control, and the design of their structure, the wide variety of self-assembling polyelectrolytes [16,21], the mild loading environment, and controlled permeability. Polyelectrolyte capsules have been used for the controlled encapsulation and release of small molecule drugs [22], enzymes [23], protein drugs [24], DNA [25], etc.

In this work, LbL polyelectrolyte capsules made from biodegradable polymers were used as an intracellular delivery system for gadolinium-doped cerium oxide nanoparticles. The research entailed the synthesis of polyelectrolyte microcapsules modified with redox-active CeGdO_2−x_ NPs, a comprehensive analysis of their physicochemical properties and biocompatibility, and the demonstration of their ability to act as an MRI contrast agent.

## 2. Materials and Methods

### 2.1. Materials

Calcium chloride (CaCl_2_, #223506), sodium carbonate (Na_2_CO_3_, #S7795), ethylenediaminetetraacetic acid disodium salt dihydrate (Na_2_EDTA, #E5134), citric acid (HOC(COOH)(CH2COOH)_2_, #C0759), cerium (III) chloride heptahydrate (CeCl_3_·7H_2_O, #22300), gadolinium(III) nitrate hexahydrate (#451134), dextran sulfate sodium salt (DS, MW ≈ 10 kDa, #D4911), 4-iodophenol (#I10201), and poly-L-arginine hydrochloride (PArg, MW ≈ 70 kDa, #P3892), were purchased from Sigma-Aldrich. All chemicals were used as received. Ultrapure water with a resistance greater than 18.2 MΩ cm^−1^ was used for all experiments. MTT reagent and Hoechst 33,342 dye were purchased from PanEKO (Moscow, Russia). A live/dead assay kit, LDH assay kit, rhodamine B isothiocyanate, and phalloidin-FITC were purchased from Thermo Fisher Scientific, Cambridge, UK.

### 2.2. CeGdO_2−x_ NPs Synthesis and Characterisation

An aqueous solution containing cerium (III) chloride and gadolinium (III) nitrate was prepared, with a total concentration of rare earth elements of 2 mM. The molar ratio of cerium to gadolinium was 4:1. An anion exchange resin in the OH form was added to the resulting solution until pH 10.0 was reached. The resulting solution was separated from the anion exchange resin by filtration and then subjected to hydrothermal treatment at 150 °C for 1.5 h, after which it was cooled to room temperature. The sol was stabilised using sodium citrate (cerium/citrate molar ratio was 1:4). Then, the pH of the sol was adjusted to 7–8 by dropwise addition of aqueous ammonia.

The absorption spectra of the CeGdO_2−x_ NPs were measured on a Lambda 950 UV/VIS spectrometer (Perkin-Elmer, Shelton, CT, USA). TEM images were acquired using a JEOL-JEM 2010 transmission electron microscope (JEOL, Tokyo, Japan). EDX analysis was performed using an FEI Inspect F microscope. The hydrodynamic diameter and the zeta potential values were measured using a Zetasizer Nano ZS analyzer (Malvern Instruments Ltd., Malvern, UK). The aggregative stability of the CeGdO_2−x_ NPs was studied by dynamic light scattering 1 h after the formation of the CeGdO_2−x_ NPs’ suspension in a phosphate buffer (pH 7.4) (PanEco, Moscow, Russia), DMEM/F12 culture medium (PanEco, Russia), and DMEM/F12 culture medium containing 10% fetal bovine serum (HyClone, Logan, UT, USA).

### 2.3. The Synthesis and Characterisation of the Capsules

Calcium carbonate (CaCO_3_) was used as a template. The CaCO_3_ synthesis was initiated by rapid mixing of equal volumes of CaCl_2_ and Na_2_CO_3_ aqueous solutions at room temperature. After intensive stirring, with a magnetic stirrer for 30 s, the precipitate was separated by centrifugation at 1000 rpms for 1 min and washed three times with water. As a result, an aqueous suspension was formed containing spherical CaCO_3_ microparticles with an average diameter of 3 to 4 µm. The first polyelectrolyte layer was deposited on the microparticles’ surface by adsorption of positively charged poly-L-arginine hydrochloride (PARg) using a 1 mg/mL^−1^ PARg solution in 0.15 M NaCl (15 min incubation and shaking). The second layer was deposited by absorbing a negatively charged sodium dextran sulfate (DS) from a 1 mg/mL^−1^ DS solution in 0.15 M NaCl (15 min incubation and shaking). The core/polyelectrolyte particles were washed three times with deionised water after each adsorption step. A colloidal solution of CeGdO_2−x_ NPs was taken at a concentration of 0.5 mg/mL. The calcium carbonate nuclei were dissolved in ethylenediaminetetraacetic acid (EDTA) for 30 min, then centrifuged and washed three times with EDTA and then three times with water. The capsule consisted of a biodegradable polyelectrolyte PArg and DS with cerium gadolinium oxide nanoparticles in the middle layer (PArg/DS) (PArg/CeGdO_2−x_ NPs) (PArg/DS).

The absorption UV spectra of the CeGdO_2−x_ NPs-loaded capsules were measured on a Perkin-Elmer Lambda 950 UV/VIS spectrometer. TEM images were acquired using a JEOL-JEM 2010 transmission electron microscope. Scanning electron microscopy (SEM) and EDX analyses were performed using an FEI Inspect F microscope. Laser scanning confocal microscopy (LSCM) images were acquired using a Leica TS laser scanning confocal microscope with a 63× f/1.4 oil immersion lens. Rhodamine B isothiocyanate (RBITC) labeled dextran was used as a fluorescent marker for the synthesis of the capsules and to study their intracellular localization.

### 2.4. Cell Culture

Cytotoxicity and cellular uptake analyses were carried out on three types of cell cultures: human osteosarcoma cells (MNNG/Hos), human adenocarcinoma cells (MCF-7), and human mesenchymal stem cells (hMSc) isolated from the dental pulp of a healthy orthodontics patient (with his written consent). All cells were deposited in the cryobank of the Theranostics and Nuclear Medicine Laboratory in ITEB RAS. Cells were cultured in a DMEM/F12 cultural medium containing 10% fetal calf serum (Cytiva, Shrewsbury, MA, USA) and a mixture of antibiotics (penicillin-streptomycin) (PanEco, Russia). The cells were cultured in 75 cm^2^ flasks (SPL, Pocheon-si, Korea) in a CO_2_ incubator (RWD, Shenzhen, China).

### 2.5. MTT Assay

Cytotoxicity was assessed using a standard MTT assay [26]. Cells were seeded in 96 well plates (SPL, Korea) at a density of 2.5 × 10^4^/cm^2^ in a DMEM/F12 culture medium (PanEko, Russia) containing 10% fetal calf serum (HyClone, Logan, UT, USA). After 8 h, capsules (1, 10, and 100 capsules per cell) were added to the cells. Then, after 24, 48, and 72 h, the medium was replaced with a solution of the MTT reagent (0.5 mg/mL). After 3 h of incubation with the MTT reagent, 100 μL of DMSO (PanEko, Russia) was added. The optical density of the formazan solution in DMSO was determined using a BioRad plate reader 680 (BioRad, Ramsey, MN, USA) at 540 nm wavelength.

### 2.6. LDH Assay

Cells were seeded in 96-well plates and cultured in an atmosphere containing 5% CO_2_, at 37 °C. Six hours after cell seeding, the medium was replaced with the similar medium containing 1, 10, or 100 capsules per cell. Triton X-100 was used as a positive control. Within 72 h after the addition of the CeGdO_2−x_ NP-loaded capsules, the level of lactate dehydrogenase in the culture medium was determined, according to the manufacturer’s protocol (The Thermo Scientific™ Pierce™ LDH Cytotoxicity Assay Kit, Cambridge, UK). Absorbance of the solution was measured at wavelengths of λ = 490 nm and λ = 640 nm, using the Microplate Reader ThermoMultiskan Ascent 96 & 384 (Thermo Fisher Scientific, Cambridge, UK).

### 2.7. Cellular Uptake

Rhodamine B isothiocyanate (RBITC)-labelled capsules were used for intracellular visualisation. The cells were seeded in 35 mm Petri dishes with a central hole (Ibidi, Fitchburg, WI, USA), at a density of 2 × 10^3^ per cm^2^. After attachment and spreading of the cells (8 h), RBITC-labelled capsules were added (10 capsules per cell) and incubated with the cells for 16 h. After that, the cells were washed three times with Hank’s solution and were stained to show actin cytoskeleton (phalloidin-FITC, Thermo Fisher Scientific, UK) and the cell nucleus (Hoechst 33342, PanEco, Russia). Micrographs were taken on a Zeiss Axiovert 200 (Zeiss, Jena, Germany) inverted microscope at a magnification of 63× with oil immersion.

### 2.8. MRI Scanning

MRI studies of CeGdO_2−x_-loaded capsules were carried out using a Bruker Clinscan 7T MRI tomograph (Bruker, Billerica, MA, USA). For the measurements, the samples were diluted with an HEPES buffer solution to concentrations ranging from 0.1 mM to 1 mM, taking into account the efficiency of the loading of the nanoparticles and the concentration of nanoparticles per capsule. The SI dependencies on TI for each concentration were plotted and the T1 relaxation time was determined by the Mathcad approximation. 1/T1 (s^−1^) values were calculated from experimentally determined T1 relaxation times. T1 relaxivity values were calculated as the tangent of the inclination angle in the dependencies of the reverse T1 relaxation time on the Gd^3+^ concentration.

### 2.9. Antioxidant Activity of CeGdO_2−x_ NPs

H_2_O_2_ was generated under X-ray irradiation of the CeGdO_2−x_ NPs’ suspensions. Irradiation was conducted using an X-ray therapeutic machine RTM-15 (Mosrentgen, Moscow, Russia) in a dose of 5 Gy, at a dose rate of 1 Gy/min, 200 kV voltage, 37.5 cm focal length, and 20 mA current. To evaluate the redox activity of the CeGdO_2−x_ NPs, the concentration of hydrogen peroxide after the X-ray exposure was measured by an enhanced chemiluminescence technique, using a luminol–4-iodophenol–peroxidase system [27]. A TRIS buffer was used to maintain a constant pH (7.2). A liquid scintillation Beta-1 counter (MedApparatura, Kyiv, Ukraine), operating in a single photon counting mode (with one photomultiplier and the coincidence scheme disengaged), was used as a highly sensitive chemiluminometer. The high sensitivity of this method enabled the registration of hydrogen peroxide at a concentration of <1 nM. The H_2_O_2_ content was determined using calibration chemiluminescence plots. The concentration of hydrogen peroxide used for the calibration was determined spectrophotometrically at 240 nm, using a molar absorption coefficient of 43.6 M^−1^·cm^−1^.

### 2.10. Statistical Data Analysis

Mean values and the standard deviation of the mean were calculated and the significance of differences between the groups was determined using the Student *t*-test.

## 3. Results

**Figure 1:**
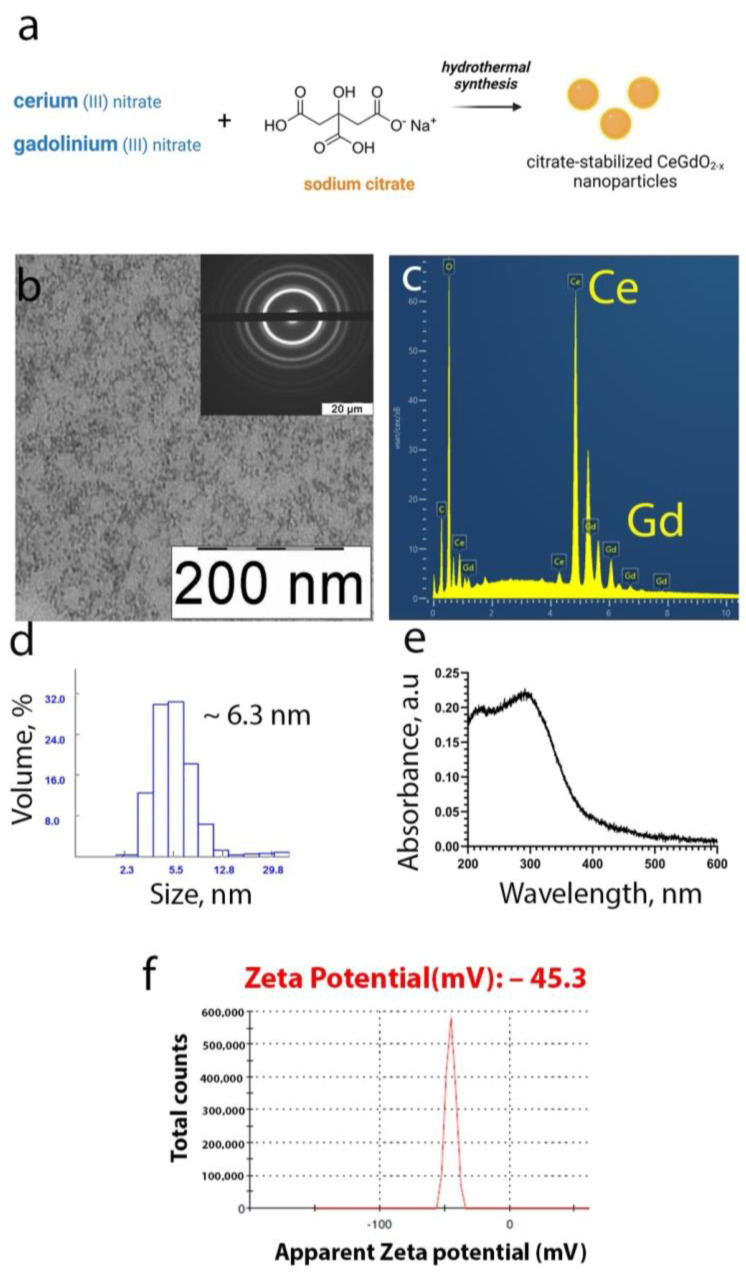
Synthesis scheme of the CeGdO_2−x_ nanoparticles (**a**), transmission electron microscopy (**b**), EDX analysis (**c**), dynamic light scattering in MQ water (**d**), UV absorbance spectrum (**e**) and zeta-potential (**f**).

For the synthesis of the CeGdO_2−x_ nanoparticles, cerium chloride and gadolinium nitrate were used (Figure 1a). Sodium citrate was used as a biocompatible stabiliser. The results of the X-ray diffraction analysis of the CeGdO_2−x_ nanoparticles indicated their ultrasmall size and crystallinity. The X-ray diffraction pattern was characteristic of the cubic Fm3m structure of nanocrystalline ceria. According to the Scherrer formula, the crystal size of the CeGdO_2−x_ nanoparticles was 2 nm. TEM data (Figure 1b) agreed well with the X-ray diffraction analysis, although individual nanoparticles could hardly be seen, due to the stabiliser layer. The chemical composition of the CeGdO_2−x_ nanoparticles was confirmed with an EDX analysis (Figure 1c). The hydrodynamic diameter of the nanoparticles in water was 6–7 nm (Figure 1d) and the UV spectrum possessed a peak characteristic of ceria at 340 nm (Figure 1e). Zeta potential of the CeGdO_2−x_ nanoparticles was −45.3 mV (Figure 1f). We analyzed the aggregation stability of CeGdO_2−x_ nanoparticles in various medium, which confirmed their high degree of colloidal stability (Table S1).

The degree of crystallinity of the cerium oxide nanoparticles plays a key role in their enzyme-like properties. It was previously shown that the synthesis of cerium oxide nanoparticles under mild conditions using hydrothermal methods makes it possible to obtain nanoparticles with a low level of crystallinity and a higher content of Ce^3+^, which ensures their high antioxidant activity. Conversely, cerium oxide nanoparticles prepared using prolonged high-temperature annealing show negligible enzyme-like properties and are unable to protect human MSCs from oxidative stress [28]. The degree of crystallinity could also affect the biodegradability of ceria NPs. For example, Plakhova et al. have showed that the anti- and pro-oxidant activity of ceria measured at different pH levels can be related to the dissolution of cerium oxide in aqueous media [29]. Thus, the conditions for the synthesis of cerium oxide nanoparticles were chosen to achieve low toxicity and high enzyme-like activity.

**Figure 2:**
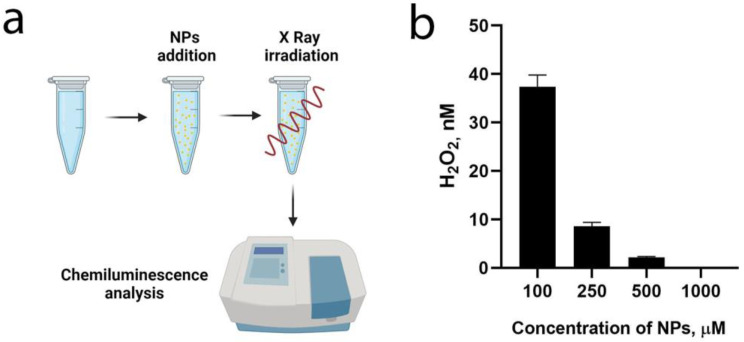
Antioxidant activity of CeGdO_2−x_ NPs under X-ray irradiation (5 Gy). Schematic representation of the experiment (**a**). Concentration of hydrogen peroxide after X-ray irradiation (total dose 5 Gy, 1 Gy per min) in CeGdO_2−x_ NPs colloid solution at pH 7.2 (**b**). The level of hydrogen peroxide was determined by the chemiluminescent method, using horseradish peroxidase.

Next, the redox activity of the synthesized nanoparticles was analyzed in different media to reveal their pronounced antioxidant properties (Figure 2). The properties were demonstrated for the CeGdO_2−x_ nanoparticles in aqueous solutions upon the X-ray irradiation (Figure 2a). The study showed that, at a concentration of nanoparticles of 250 μM, the amount of the hydrogen peroxide formed was reduced by a factor of three in comparison with the control experiment. At a concentration of nanoparticles of 1 mM, the level of hydrogen peroxide in the sol after the X-ray irradiation was equal to zero (Figure 2b). These data are consistent with the authors’ earlier data on the antioxidant properties of CeO_2_ nanoparticles, which showed that, at the concentration of 10^−5^ M, they were able to effectively reduce the generation of radiation-induced hydrogen peroxide [30]. The redox properties of nanoceria enables it to work as a scavenger of reactive oxygen species (ROS) and free radicals, preventing the development of radiation-induced damage to cells, organs, and whole organisms [31,32,33,34,35]. It has previously been shown that nanoceria is capable of inactivating a wide range of radicals and ROSs, including the hydroxyl radical [36], nitroxyl radical [37], singlet oxygen [38], and superoxide anion radical [39]. Nanoceria is generally considered as a unique inorganic nanozyme with a wide spectrum of scavenging and anti-inflammatory activity, which makes it promising in the treatment of various diseases.

**Figure 3:**
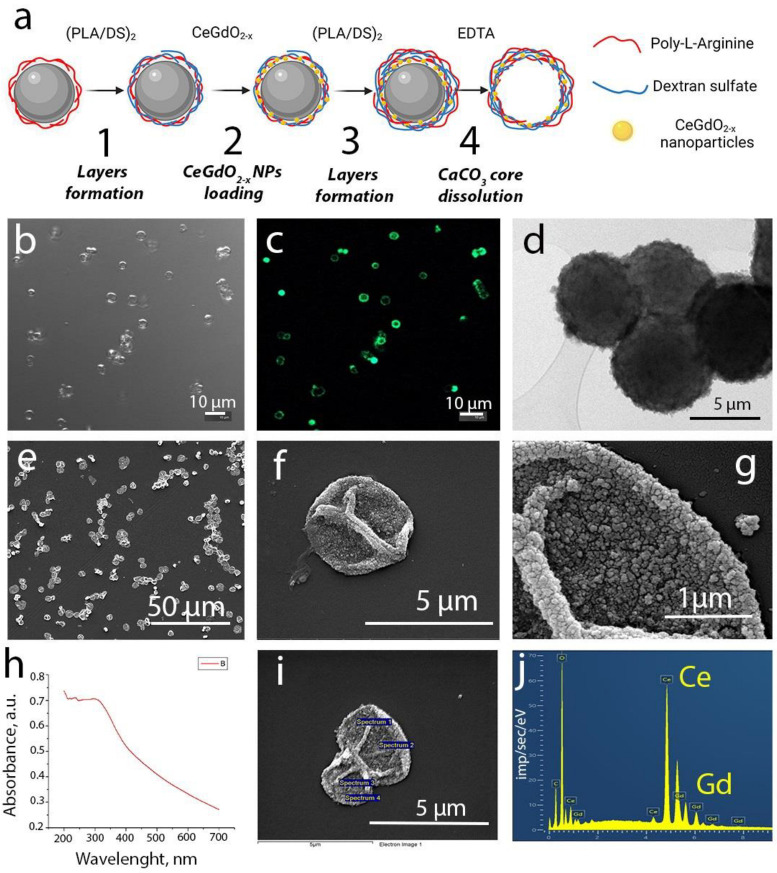
Scheme of CeGdO_2−x_ NPs-loaded microcapsules synthesis (**a**), LSCM (**b**,**c**), TEM (**d**) and SEM (**e**–**g**) images. UV–visible spectrum (**h**) and EDX analysis (**i**,**j**).

The method of layer-by-layer adsorption of differently charged polyelectrolytes was used for the synthesis of the microcapsules, as shown in Figure 3a. The efficiency of LBL capsule formation was confirmed by zeta potential analysis after deposition of each polyelectrolyte layer (Figure S1). The integration of CeGdO_2−x_ nanoparticles into the middle layer was carried out by mixing the sol of the nanoparticles with a negatively charged polyelectrolyte (dextran sulfate). Interestingly, the integration of CeGdO_2−x_ nanoparticles in the capsules provided them a luminescent property, which was revealed using a confocal microscope (Figure 3b,c). The reason for the bright glow was not investigated since this was outside the scope of the work. The study of the microcapsules by transmission electron microscopy confirmed the effective sorption of nanoparticles, which is indicated by dark areas throughout the microcapsule (Figure 3d). The integration of nanoparticles into the structure of microcapsules was also confirmed by scanning electron microscopy, where nanoparticle aggregates can be observed at high magnification (Figure 3e–g). Energy dispersive spectroscopy (EDX) confirmed the presence of cerium and gadolinium in the structure of the synthesized microcapsules (Figure 3i,j). The UV-visible spectrum of the synthesized microcapsules is shown in Figure 3h, which shows the peak characteristic of nanocrystalline ceria.

**Figure 4:**
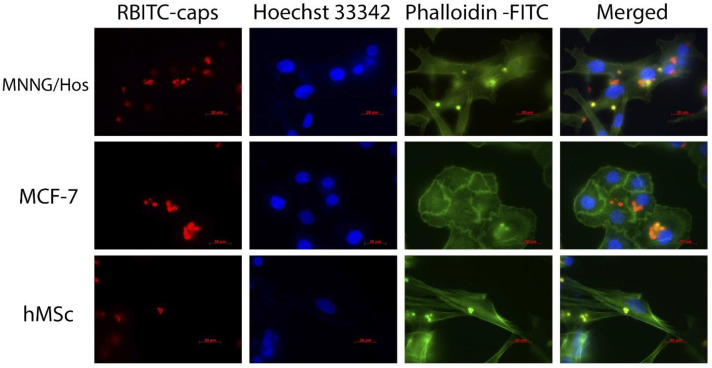
Cellular uptake of RBITC-labelled CeGdO_2−x_ NPs-loaded LbL microcapsules. Different types of cells (MNNG/Hos, MCF-7 and hMsc) were stained by phalloidin-FITC (green, actin cytoskeleton staining) and Hoechst 33,342 (blue, cell nucleus staining). Scale bar is 20 µm.

The polyelectrolyte microcapsules can be taken up by various types of cells, including non-phagocytic cells. The process of endocytosis of the polyelectrolyte microcapsules has been well studied, and has been demonstrated in both in vitro systems and in vivo models. Prior to this, the mechanisms of the penetration of the capsules into cells with the help of pharmacological inhibitors were studied [40]. It was shown that the main mechanism of the penetration of the capsules into human hepatocellular carcinoma HepG2 cells is endocytosis. At the same time, the deformability/stiffness of the microcapsules govern the rate of both endocytosis and exocytosis. It should be noted, however, that micron-sized capsules can be absorbed not only by phagocytic cells, but also by many others [41]. The shape of the microcapsules influences the penetration efficiency into smooth muscle cells and macrophages, through bending and faster internalization [42]. In this study, spherical capsules were used, and this shape facilitated a fairly effective uptake by cells of various types, including human hMScs (Figure 4). It should be noted that the loading of capsules with nanoparticles led to a change in the structure of the outer shell of the microcapsules and an increase in its roughness (Figure 3d), which provided additional sites for interaction with the cell membranes, and thus increased the absorption efficiency. It has been shown that human osteosarcoma cells (MNNG/Hos line) and human adenocarcinoma cells (MCF-7 line) also efficiently took up the hybrid microcapsules containing gadolinium-doped cerium oxide nanoparticles.

Thus, the integration of redox-active nanoparticles into the shell of microcapsules made it possible to simultaneously deliver a much higher concentration of nanoparticles into the cells compared to a bare sol of nanoparticles.

**Figure 5:**
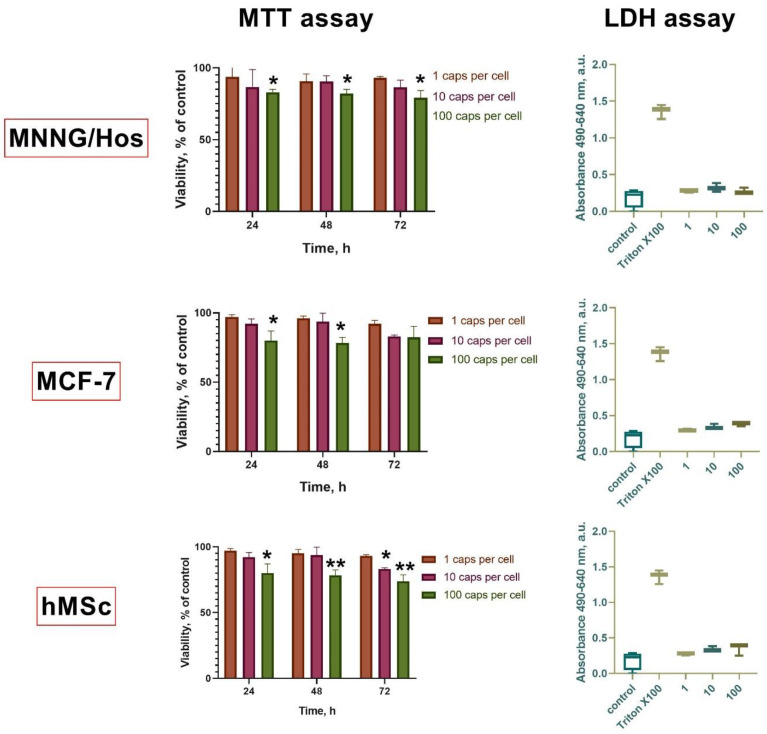
MTT assay 24, 48, and 72 h after incubation with microcapsules (1, 10 or 100 per cell), LDH assay 72 h after incubation with CeGdO_2−x_ microcapsules (1, 10, or 100 per cell). * *p* ≤ 0.05, ** *p* ≤ 0.01.

Next, a comprehensive analysis was carried out of the cytotoxicity of the synthesized microcapsules on three types of cell cultures: human osteosarcoma MNNG/Hos cells, human adenocarcinoma MCF-7 cells, and mesenchymal stem cells isolated from human dental pulp. Two tests were used for analysis: an MTT test to analyze the level of dehydrogenase activity, and an LDH test to detect dead (lysed) cells (Figure 5). It was found that the capsules at the highest concentration (100 capsules per cell) after 24 h of co-incubation caused a significant decrease in the level of viability for all types of cell cultures studied. Further incubation (48 and 72 h) confirmed the negative effect of the microcapsules at high concentrations; the viability of the cells was reduced to 80% for human MSCs and to 84% for MNNG/Hos cells. However, low concentrations of capsules (1 or 10 capsules per cell) did not cause a statistically significant decrease in the cell viability, while a downward concentration trend was observed for all the cell types. The most sensitive to the contact with the capsules were human MSCs. At the same time, the analysis of the level of free lactate dehydrogenase (LDH assay) did not reveal statistically significant differences, which presumably indicates that the capsules affect the cells’ metabolic profile and their proliferative and migratory activity, but do not cause any damage to cell membranes or cause their death even at high concentrations. Studies of the toxicity of magnetic capsules to human MSCs synthesized from poly(allyl)amine hydrochloride and poly(styrene) sulfonate were reported recently [43]. It has been shown that, at concentrations of less than 100 microcapsules per cell, they were not toxic and can be effectively internalized. It has also been shown that hMSCs can be efficiently loaded with microcapsules without damaging the cell’s structural integrity [44]. The LbL microcapsules were not shown to reduce cell viability but changed the structure of the actin cytoskeleton of cells [45]. Thus, it can be concluded that the synthesized microcapsules have a high level of biocompatibility.

**Figure 6:**
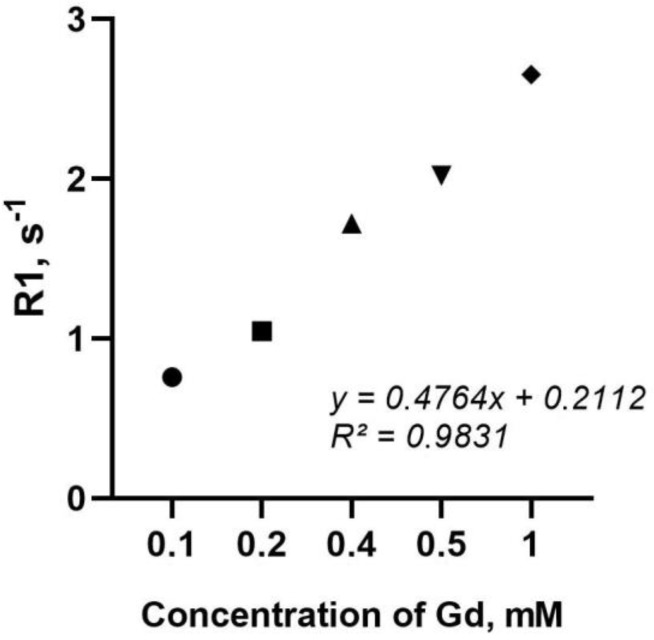
*R*_1_-relaxation rates for CeGdO_2−x_ NPs-loaded LbL capsules at different concentrations of Gd.

The next task was to find out how the integration of the nanoparticles into the structure of the capsules affects the relaxation rate in MRI measurements (Figure 6). Previously, the authors demonstrated the MRI contrast ability of dextran-stabilized gadolinium-doped cerium oxide nanoparticles [46], which amounted to 3.6 mM·s^−1^. The analysis of the relaxation rate of the synthesized LbL capsules loaded with CeGdO_2−x_ NPs demonstrated their lower relaxation rate (2.75 mM·s^−1^), which could be due to the aggregation of CeGdO_2−x_ NPs in the structure of the polyelectrolyte matrix and the low access level to water molecules (electron spins), or the possible partial loss of the nanoparticles during the multistage synthesis of the composite capsules, as previously shown [47]. At the same time, it should be noted that the relaxation rate for such CeGdO_2−x_ loaded microcapsules is significantly inferior to commercial preparations [48]. Given that, after the internalization of such microcapsules by the cells, the loaded nanoparticles were released and distributed in the cytoplasm, it can be assumed that the relaxation rate parameter should increase. There are different ways to incorporate gadolinium compounds into the capsules, e.g., they can be loaded into the internal compartment of the capsules. Meanwhile, the question of how to fix a gadolinium compound in the center of a capsule instead of allowing it to move freely inside a capsule still remains unresolved. The second approach is to incorporate gadolinium compounds into the capsule shell; this can be achieved by conjugating contrast agents to a polyelectrolyte, or by using nanoparticles with a high surface charge, which will bind to an oppositely charged polyelectrolyte. The latter approach was used in this study. It is very important to provide linking spacers that have good rigidity to bridge the ligand and the polymer. It is also necessary to take into account the influence of the template solvent, since the pH value of such a solvent can affect the properties of the loaded paramagnetic nanoparticles, the degree of their aggregation, and possible dissolution. Thus, the use of paramagnetic particles makes it possible to create MRI agents based on LbL capsules; however, it is necessary to ensure certain conditions for the integration of nanoparticles into the capsule structure are met, and to select conditions for the synthesis and localization of nanoparticles, which governs their MRI contrasting property.

## 4. Conclusions

This paper has demonstrated the fabrication of biodegradable microcapsules with CeGdO_2−x_ NPs nanoparticles in their shell with an MRI contrasting property. The new microcapsules had a size of 3–4 µm. A comprehensive analysis of their physicochemical properties has confirmed the effective loading of nanoparticles into the structure of the capsules. Furthermore, the synthesized capsules effectively penetrated both normal and cancer cells, being localized in the cytoplasm after the internalization. The microcapsules were not toxic at concentrations below 100 capsules per cell. The combination of MRI imaging and the redox properties of the capsules opens up possibilities for their use as theranostic agents and drug carriers with an easy to trace localization.

## Data Availability

Data is contained within the article or Supplementary Materials.

## Supplementary Materials

The following supporting information can be downloaded at: https://www.mdpi.com/article/10.3390/polym15183840/s1, Table S1: The aggregative stability of CeGdO_2−x_ NPs via DLS study in different medium. Figure S1: Changes in zeta potential of CeGdO_2−x_ loaded capsules while layer by layer assembly. Layer 1st, 3rd, 5th: positively charge (poly-l- arginine). Layer 2nd, 4th, 6th: negatively charge (2nd and 6th dextran sulphate, 4th—citrate-stabilized CeGdO_2−x_ NPs) applied on calcium carbonate template.
