# Supplementary material for: Hybrid Polyelectrolyte Capsules Loaded with Gadolinium-Doped Cerium Oxide Nanoparticles as a Biocompatible MRI Agent for Theranostic Applications
Source: Polymers (Basel). 2023 Sep 21;15(18):3840. doi: 10.3390/polym15183840 (PMC10536467; doi:10.3390/polym15183840)
Supplement: Supplementary file 1 [file polymers-15-03840-s001.zip › polymers-2597845-supplementary.pdf]

Article

Hybrid polyelectrolyte capsules loaded with gadolinium-doped cerium oxide nanoparticles as a biocompatible MRI agent for theranostic applications

Danil D. Kolmanovich[1], Nikita N. Chukavin[1], Irina V. Savintseva[1], Elena A. Mysina[1], Nelli R. Popova[1], Alexander E. Baranchikov[2*], Madina M. Sozarukova[2], Vladimir K. Ivanov[2] and Anton L. Popov[1*]

[1]Institute of Theoretical and Experimental Biophysics, Russian Academy of Sciences, Pushchino 142290, Moscow Region, Russia

[2] Kurnakov Institute of General and Inorganic Chemistry of the Russian Academy of Sciences, Moscow 119991, Russia

* Correspondence: a.baranchikov@yandex.ru (A.E.B.); antonpopovleonid@gmail.com (A.L.P.)

Table S1. The aggregative stability of $CeGdO_{2-x}$ NPs via DLS study in different medium

	mQ	PBS (pH 7.4)	NaCl 0.9%	DMEM/F12	DMEM/F12+10% FBS
Z-среднее (d.nm)	**6.4**	**10.5**	**15.6**	**60.12**	**8.9**
PDI	**0.231**	**0.366**	**0.433**	**0.372**	**0.323**

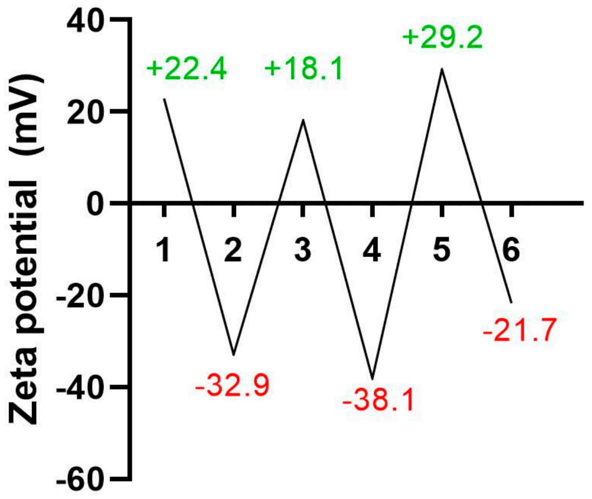

Figure S1. Changes in zeta potential of $CeGdO_{2-x}$ loaded capsules while layer by layer assembly. Layer 1st, 3rd, 5th: positively charge (poly-l- arginine). Layer 2nd, 4th, 6th: negatively charge (2nd and 6th dextran sulphate, 4th – citrate-stabilized $CeGdO_{2-x}$ NPs) applied on calcium carbonate template
